# The ReInforcement of adherence via self-monitoring app orchestrating biosignals and medication of RivaroXaban in patients with atrial fibrillation and co-morbidities: a study protocol for a randomized controlled trial (RIVOX-AF)

**DOI:** 10.3389/fcvm.2023.1130216

**Published:** 2023-05-24

**Authors:** Minjae Yoon, Jin Joo Park, Taeho Hur, Cam-Hao Hua, Chi Young Shim, Byung-Su Yoo, Hyun-Jai Cho, Seonhwa Lee, Hyue Mee Kim, Ji-Hyun Kim, Sungyoung Lee, Dong-Ju Choi

**Affiliations:** ^1^ Division of Cardiology, Department of Internal Medicine, Seoul National University Bundang Hospital, Seoul National University College of Medicine, Seongnam, Republic of Korea; ^2^ Department of Computer Science and Engineering, Kyung Hee University, Yongin, Republic of Korea; ^3^ Division of Cardiology, Department of Internal Medicine, Severance Hospital, Yonsei University College of Medicine, Seoul, Republic of Korea; ^4^ Department of Internal Medicine, Yonsei University Wonju College of Medicine, Wonju, Republic of Korea; ^5^ Department of Internal Medicine, Seoul National University Hospital, Seoul National University College of Medicine, Seoul, Republic of Korea; ^6^ Division of Cardiology, Department of Internal Medicine, Cardiovascular Center, Keimyung University Dongsan Hospital, Daegu, Republic of Korea; ^7^ Division of Cardiology, Department of Internal Medicine, Chung-Ang University Hospital, Seoul, Republic of Korea; ^8^ Cardiovascular Center, Dongguk University Ilsan Hospital, Goyang, Republic of Korea

**Keywords:** atrial fibrillation, NOAC, drug adherence, mobile application, mobile health

## Abstract

**Background:**

Because of the short half-life of non-vitamin K antagonist oral anticoagulants (NOACs), consistent drug adherence is crucial to maintain the effect of anticoagulants for stroke prevention in atrial fibrillation (AF). Considering the low adherence to NOACs in practice, we developed a mobile health platform that provides an alert for drug intake, visual confirmation of drug administration, and a list of medication intake history. This study aims to evaluate whether this smartphone app-based intervention will increase drug adherence compared with usual care in patients with AF requiring NOACs in a large population.

**Methods:**

This prospective, randomized, open-label, multicenter trial (RIVOX-AF study) will include a total of 1,042 patients (521 patients in the intervention group and 521 patients in the control group) from 13 tertiary hospitals in South Korea. Patients with AF aged ≥19 years with one or more comorbidities, including heart failure, myocardial infarction, stable angina, hypertension, or diabetes mellitus, will be included in this study. Participants will be randomly assigned to either the intervention group (MEDI-app) or the conventional treatment group in a 1:1 ratio using a web-based randomization service. The intervention group will use a smartphone app that includes an alarm for drug intake, visual confirmation of drug administration through a camera check, and presentation of a list of medication intake history. The primary endpoint is adherence to rivaroxaban by pill count measurements at 12 and 24 weeks. The key secondary endpoints are clinical composite endpoints, including systemic embolic events, stroke, major bleeding requiring transfusion or hospitalization, or death during the 24 weeks of follow-up.

**Discussion:**

This randomized controlled trial will investigate the feasibility and efficacy of smartphone apps and mobile health platforms in improving adherence to NOACs.

**Trial registration:**

The study design has been registered in ClinicalTrial.gov (NCT05557123).

## Introduction

For the management of patients with atrial fibrillation (AF), stroke prevention with oral anticoagulants is central to guideline-recommended management (1–3). Previous large randomized controlled trials for AF have demonstrated improved safety and comparable effectiveness of non-vitamin K antagonist oral anticoagulants (NOACs) compared with warfarin (4–8). Also, in contrast to warfarin, NOACs have minimal drug or food interactions and predictable effectiveness with fixed dosing without the need for laboratory monitoring. Because of these benefits, NOAC use is expanding globally (9, 10), and the current guidelines favor NOACs over warfarin for stroke prevention in AF (1, 2).

However, there are issues with the relatively short half-life of NOACs. When doses are missed, there is a higher risk of a prothrombotic state (11). Therefore, during NOAC therapy, constant medication compliance is crucial to sustain the anticoagulation's effect on stroke prevention (12, 13). Failed persistence or poor drug adherence to NOACs may increase the risk of stroke, which may result in rising overall health care costs (14–17).

Recently, Desteghe et al. showed that telemonitoring with or without feedback resulted in higher NOAC adherence (18). However, monitoring and feedback by health care providers have high costs and require more infrastructure and manpower and, thus, are challenging to incorporate in an actual clinical setting. Currently, smartphones are available to most of the general population at affordable costs. With the advent of mobile technology and advances in artificial intelligence (AI), mobile-based feedback algorithms may be promising strategies to improve medication adherence with easy distribution (19). Although there are some debates over their efficacy, smartphone applications (apps) are currently acknowledged as an effective method for improving drug adherence (20–28). Smartphone apps can transmit push alarms or notifications to remind the patient to take their medication, which may function to increase drug adherence (29). There are some previous studies regarding mobile apps for improving drug adherence of NOACs (30, 31). However, these studies had a small number of participants and limited application functions. Considering the low drug adherence of NOACs in patients with AF in the real world (13, 17, 32), we developed a mobile health platform to provide an alert for drug intake, visual confirmation of drug administration, and a list of medication intake history. This study aims to evaluate whether this smartphone app-based intervention will increase drug adherence compared with usual care in a large population of patients with AF requiring NOACs.

## Methods and analysis

### Study design

The ReInforcement of adherence Via self-monitoring app Orchestrating biosignals and medication of RivaroXaban in patients with Atrial Fibrillation and co-morbidities (RIVOX-AF) study is a prospective, randomized, open-label, nationwide, multicenter trial to evaluate the efficacy of smartphone apps (MEDI-app) in improving drug adherence to NOACs in patients with AF. Thirteen tertiary university hospitals in South Korea will participate in this study. Enrollment began in March 2022, is currently ongoing, and is expected to be completed in late 2023. The study design has been registered at ClinicalTrials.gov (NCT05557123).

### Study population

We will enroll patients with AF aged 19 years or older with one or more comorbidities, including heart failure, myocardial infarction, stable angina, hypertension, or diabetes mellitus. Patients can be enrolled 3 months after myocardial infarction or percutaneous coronary intervention. Rivaroxaban (Rivoxaban, Samjin Pharm, Seoul, Korea) will be administered to the participants at an open-label dosage of 20 mg once daily. Patients using other NOACs can be enrolled after swiching to Rivoxaban. The dosage will be reduced to 15 mg or 10 mg once daily if creatinine clearance is 15–49 ml/min or at the physician's discretion. Since smartphone usage is essential in this study, participants should be proficient in the use of smartphones and be able to understand and follow Korean instructions for using the program. Patients with severe chronic kidney disease (creatinine clearance <15 ml/min), moderate or severe mitral valve stenosis, or a history of mitral valve replacement or repair will be excluded. The detailed inclusion and exclusion criteria are listed in Table 1.

**Table 1:** Inclusion and exclusion criteria.

***Inclusion criteria:***
1. Patients with AF aged 19 years or older with one or more comorbidities including heart failure, myocardial infarction, stable angina, hypertension or diabetes mellitus (Patients can be enrolled 3 months after myocardial infarction or percutaneous coronary intervention). 2. Patients with AF who are taking or initiating newly-prescribed rivaroxaban 3. Patients who can use a smartphone and are fluent in Korean 4. Patients who voluntarily consent to participate in this clinical trial
***Exclusion criteria:***
1. Creatinine clearance less than 15 ml/min 2. Moderate or severe mitral stenosis 3. Previous history of mitral valve replacement or mitral valve repair 4. Previous history of alcohol or drug abuse 5. Patients who are judged as both legally and psychologically inadequate to participate in the clinical study by the investigator 6. Patients who have participated in clinical studies with other investigational drug products within 4 weeks prior to screening 7. Patients unwilling to participate in the clinical study

AF, atrial fibrillation.

At baseline, sex, age, and comorbidities will be evaluated in all participants. The CHA_2_DS_2_-VASc score for stroke risk prediction will be calculated by the summation of all assigned points: one point each for congestive heart failure (C), hypertension (H), age between 65 and 74 years (A), diabetes mellitus (D), vascular disease (V), and female sex (Sc) and 2 points each for a history of stroke/transient ischemic attack/thromboembolism (S_2_) or age ≥75 years (A_2_) (1, 33).

### Patient recruitment and randomization

Any patient who comes to the clinic for AF management will be considered a potential participant. After a comprehensive interview, eligible participants will be asked to provide written consent for participation in the trial. A baseline survey covering demographics and cardiovascular comorbidities will be given to eligible participants (Table 2). These data will be collected by a research nurse, who will re-interview the patient and refer to the doctor's notes. Then, using a web-based central randomization service (http://matrixmdr.com), eligible participants will then be randomly assigned to either the intervention group (MEDI-app) or the conventional treatment group in a 1:1 ratio. It will not be possible to blind the participants to group allocation owing to the nature of the intervention.

**Table 2:** Standard protocol items: Recommendation for interventional trials (SPIRIT) check list.

	STUDY PERIOD			
	Enrolment	Allocation	Post-allocation	Close-out
TIMEPOINT	Day -28 to 0	Visit 1 (day 0)	Visit 2 (12 weeks)	Visit 3 (24 weeks)
ENROLMENT:				
Eligibility screen	X			
Informed consent	X			
Allocation		X		
INTERVENTIONS:				
Intervention (MEDI-app)		♦——————————————————————————♦		
Control (Conventional Treatment)		♦——————————————————————————♦		
ASSESSMENTS:				
Baseline characteristics	X			
Drug adherence		X	X	X
Secondary endpoints			X	X

### Intervention (smartphone app-conditioned feedback) and follow-up

**Figure 1:**
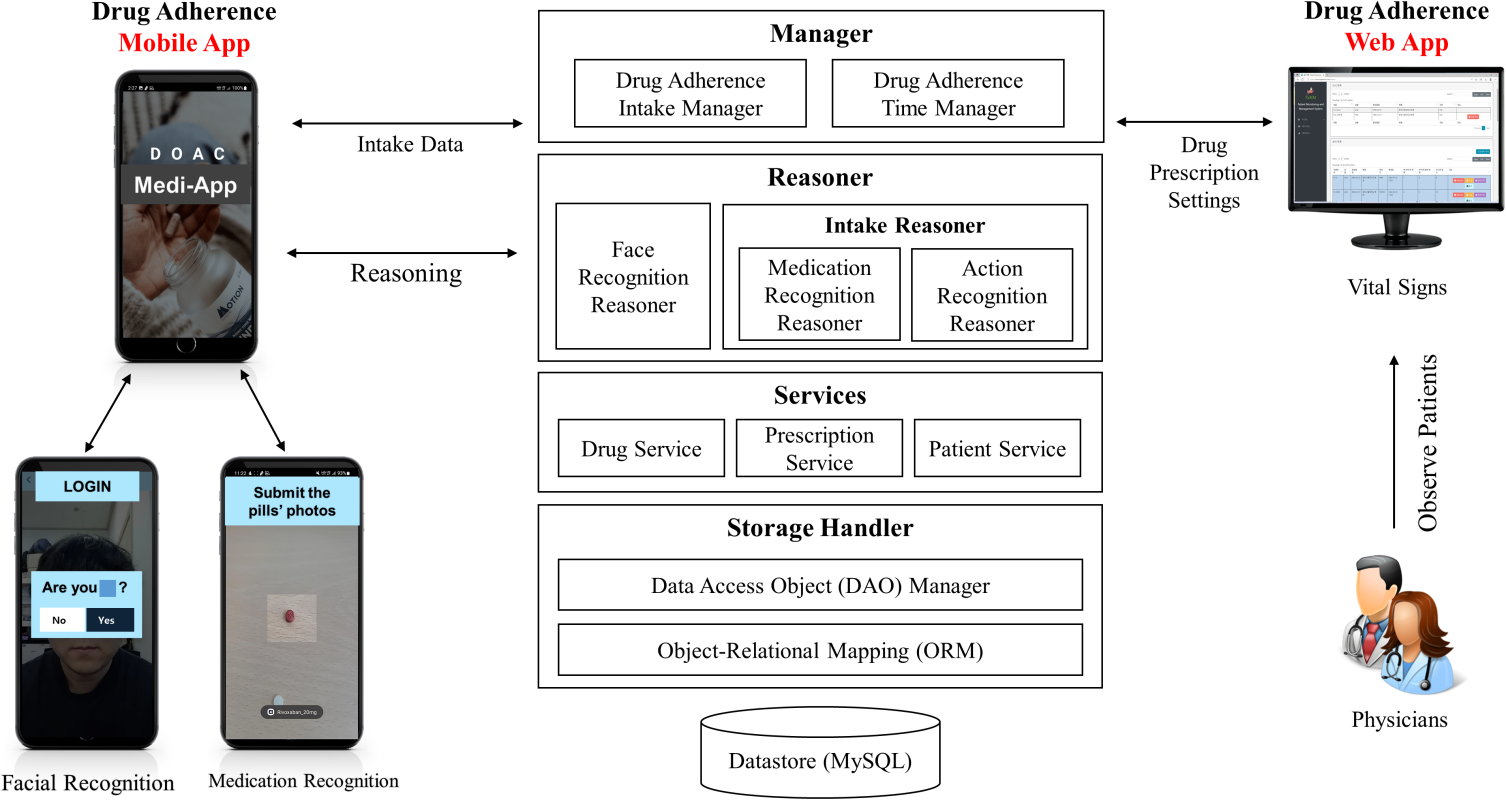
System architecture and structural workflow.

**Figure 2:**
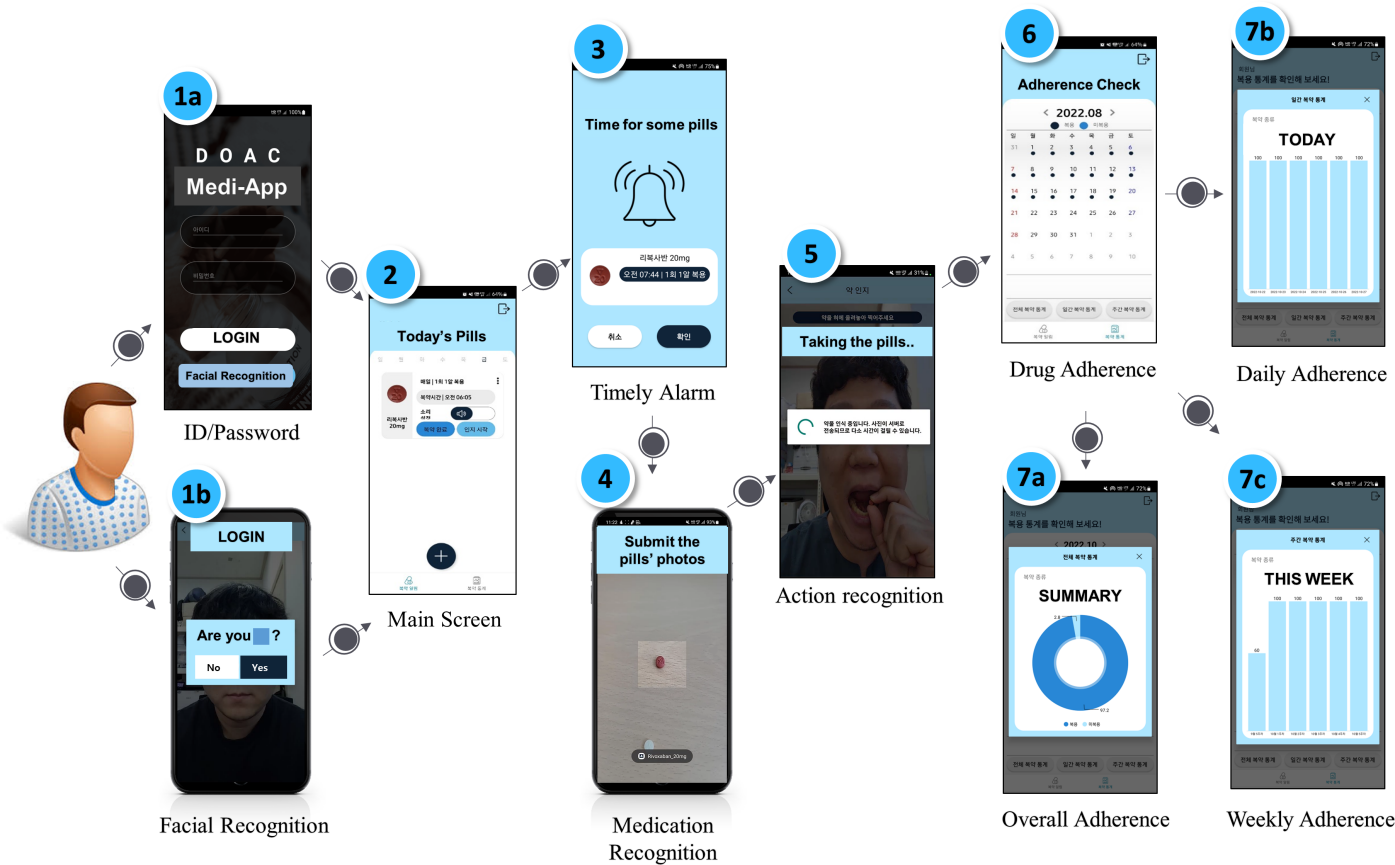
Operating system and sample display of the smartphone app. Workflow of facial login, medication recognition, action recognition, and drug adherence are shown.

We developed a mobile application (MEDI-app) to improve NOAC adherence in patients with AF. Participants in the intervention group will install the study app provided by a research nurse on their smartphones (Figure 1). Because the app is not an open application available to the public, the control group cannot access the application. The workflow, operating system, and display of the smartphone application are shown in Figures 1, 2. The patient can use facial recognition to log in to the app (Supplementary Figure S1). The design, development, and utilization details of the physician-oriented web and patient-oriented smartphone app are described in the online Supplementary material.

The research coordinator will enter the information on rivaroxaban administered to the patient into the mobile application and will set the alarm initially. The participants will be able to change the time of the alarm for rivaroxaban. When the alarm sounds at a pre-specified time, the patient will be reminded to take rivaroxaban. The app also determines whether the patient takes the medication through medication and action recognition software (Supplementary Figure S2). To elaborate, the first step is medication recognition, which determines whether the patient is taking the correct pill. A deep learning model of medication recognition will be exported after the completion of the training phase to perform real-time inference on the server from a picture captured by the camera on the patient's smartphone to match the name, shape, and color of the pill. In the second step, the app will visually confirm if the patient has taken the pill through action recognition via the smartphone camera by watching the patient put the medicine into their mouth through the camera.

If the patients do not confirm taking the medication at the scheduled time, the app will send an alert message to ensure that the patients take the medicine until 2 h after the set drug intake time. If the patient does not comply with the pill intake procedure within these 2 h, it is assumed that the patient did not take the pill, and the app will send non-intake data to the server accordingly. As an alternative, patients can also confirm that they have taken the medicine by pressing the intake button within a day, rather than using action recognition via the camera. This information can be updated on the server. Data regarding the time of rivaroxaban intake will be stored daily, and patients can check the list of drug adherence per week or month on a calendar through the app. The dashboard system is designed to show the physicians the history and number of days that the participants confirmed taking their medication (Supplementary Figure S3). In this study, the app will be initially configured only for the rivaroxaban dose, frequency, and pill supply.

**Figure 3:**
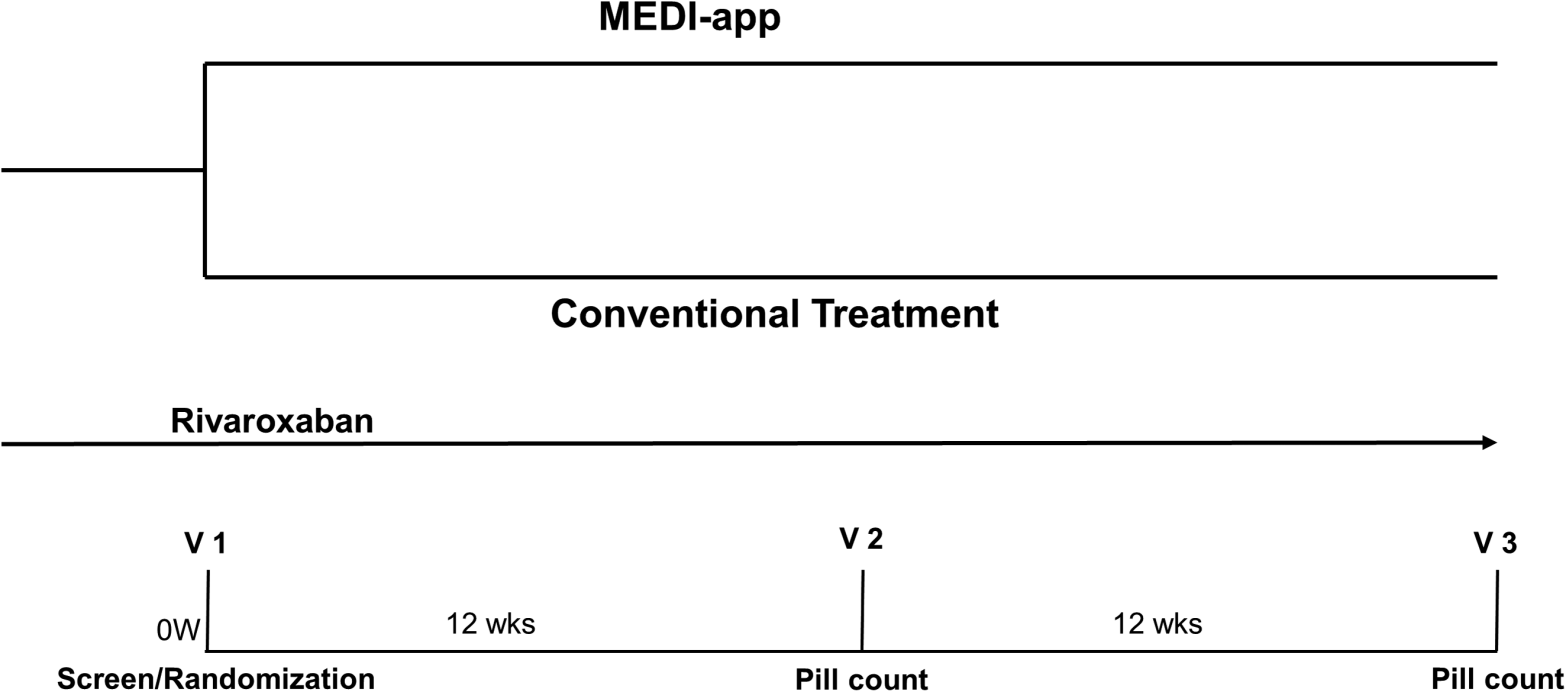
Study design of RIVOX-AF. Patients taking rivaroxaban will be randomized to either an application-conditioned feedback group or a conventional treatment group. Follow-up visits will be scheduled at 12 (visit two) and 24 (visit three) weeks after randomization.

The control group will receive only standard care which is recommended in the guidelines, including education on the disease and the importance of medication compliance by physicians at each scheduled visit. No other specific interventions will be performed. Follow-up visits will be scheduled at 12 (visit two) and 24 (visit three) weeks after randomization (Figure 3).

### Study outcomes

The primary endpoint of the study is adherence to rivaroxaban at 12 and 24 weeks. Drug adherence will be evaluated with the “pill count” measurements. The patients brought the remaining tablets to each scheduled visit, and trained and certified research nurses counted the number of returned drugs and calculated drug adherence as follows:

$$\begin{array}{rl} & \mathrm{Drug}\;\mathrm{adherence}\;=\\ & \frac{\mathrm{number}\;\mathrm{of}\;\;\mathrm{pills}\;\mathrm{dispensed}-\mathrm{number}\;\mathrm{of}\;\mathrm{pills}\;\mathrm{returned}}{\mathrm{number}\;\mathrm{of}\;\mathrm{days}\;\mathrm{between}\;\mathrm{dispensing}\;\mathrm{date}\;\mathrm{and}\;\mathrm{follow}-\mathrm{up}\;\mathrm{date}} \end{array}$$

In addition, we will evaluate the concordance of drug adherence by pill count measurements and app-calculated indices.

The key secondary endpoints are clinical composite endpoints, including systemic embolic events, stroke, major bleeding requiring transfusion or hospitalization, or death during the 24 weeks of follow-up. Other secondary endpoints include (1) major adverse cardiac and cerebrovascular events and hospitalization, (2) thromboembolism (stroke, transient ischemic attack, pulmonary embolism), (3) major bleeding requiring hospitalization or transfusion, or (4) minor bleeding. To evaluate the feasibility of the smartphone app, additional analysis will be conducted regarding the frequency of smartphone app use.

### Sample size and statistical analysis

To date, no large studies have evaluated the use of a smartphone app to improve adherence to NOACs using pill count measurements. Thus, precise sample size calculation is not possible. We assumed that drug adherence would be 91% in the control group and 95% in the intervention group with a standard deviation of 17%, considering previous studies (30, 34, 35). With a two-tailed alpha of 0.05, power of 0.95, and a dropout rate of 10%, 1,042 patients (521 patients in the intervention and 521 patients in the control groups) are required.

Categorical variables will be reported as frequencies (percentages), and continuous variables will be expressed as means ± standard deviations or medians with interquartile ranges. Categorical variables will be compared using Pearson's chi-square test or Fisher's exact test, and continuous variables will be compared using Student's *t*-test or the Mann–Whitney *U* test.

Intention-to-treat analyses will include all randomized patients. Efficacy endpoints will be mainly analyzed using the full analysis set, which includes all randomly assigned participants who underwent at least one assessment of the primary endpoint. We will also perform a per-protocol sensitivity analysis, including all patients who completed the study protocol. In the subgroup analyses, we will evaluate the differential effects of the intervention on the primary outcomes with respect to sex, age and other comorbidities.

All tests will be two-tailed, and a *P*-value <0.05 will be considered statistically significant. Statistical analyses will be performed using R version 4.2.0 (The R Foundation for Statistical Computing, Vienna, Austria).

## Discussion

This randomized controlled trial will investigate the feasibility and efficacy of smartphone apps and mobile health platforms in improving adherence to NOACs. Additionally, current study will also evaluate the benefits of app-based interventions in the reduction adverse clinical events related to AF. We believe that this prospective, randomized, large, multicenter trial will be helpful in developing public health strategies to improve NOAC adherence through smartphone use.

Poor drug adherence to NOACs may increase the risk of stroke, which may result in rising overall health care costs (14–17). Therefore, consistent drug adherence is important to maintain the effect of anticoagulation during NOAC therapy. Recently, Thakkar et al. demonstrated that mobile phone text messaging improves medication adherence in chronic disease (36). Also, Desteghe et al. showed that telemonitoring with or without feedback resulted in higher NOAC adherence (18). However, text messaging, telemonitoring, and feedback by health care providers require time, financial expenditure, and personnel and, thus, are challenging to incorporate in a real clinical setting. Today, smartphones are available to most of the general population at affordable costs. With the advent of mobile technology and advances in AI, mobile-based feedback algorithms may be promising strategies to assist in the self-management and improvement of drug adherence in chronic diseases, including AF, using cost-effective and accessible methods. We believe that our approach of using a smartphone app would be an efficient way to improve drug adherence at a minimum cost.

There have been some previous studies on mobile apps for improving drug adherence to NOACs (30, 31). However, these studies had a limitation. The studies by Labovitz et al. (30) and Turakhia et al. (31) enrolled only small numbers of patients, 28 and 139 patients, respectively. In addition, their smartphone app functions were different from ours; our mobile app and mobile health platform functions include an alarm for drug intake, visual confirmation of drug administration through a camera check, and presentation of a list of medication intake history using AI and action recognition. The study by Turakhia et al. (31) failed to determine the primary and secondary drug adherence outcomes. We believe that these heterogeneous and diverse functions of mobile interventions could affect the benefits and outcomes of mobile apps, especially for improving medication adherence.

This study's objective differs from that of the ongoing trial at our institution. In the Adhere-App study (35), patients received an alarm to take their medication and measure heart rate (HR) and blood pressure (BP) according to a pre-specified schedule on the smartphone app. The automatic BP machine is connected to the smartphone via Bluetooth, and the measured BP and HR are automatically updated on the smartphone app. The Adhere-App study is designed for the context of active participation in vital sign measurement; using a smartphone app-based feedback system could help patients become more aware of their underlying condition. In turn, this may affect their self-care habits and increase drug adherence. In the current RIVOX-AF study, we aim that smartphone app-based intervention including an alert for drug intake, visual confirmation of drug administration, and a list of medication intake history would increase drug adherence

### Limitations and strengths

This study has several limitations. First, the results of our trial may not be applicable to patients who are not capable of using smartphones, such as the elderly, because we will only enroll participants who have smartphones and are able to use them. As a large proportion of patients with AF are elderly and have difficulties using smartphones, excluding them from participation may lead to bias and study limitations. Second, the participants who consent to participate in the study may be more concerned with their own health. As a result, their medication adherence may be greater than that of the general AF population. Third, our sample size calculation may not be accurate because no prior large-scale trial has evaluated the use of smartphone apps to improve NOAC adherence. It is also possible that our estimates of drug adherence may overestimate the actual adherence, considering the findings of a previous study (17). In addition, the sample size has been calculated to determine differences in drug adherence; it is not powered to reveal any differences in adverse clinical events. Fourth, the follow-up period of this study was limited to 24 weeks. Although longer follow-up periods may provide a more reliable data on adherence, the follow-up period in our study was decided on the basis of manpower and cost.

Despite these limitations, this study is a nationwide, multicenter randomized control trial with a relatively large number of participants compared with previous studies (30, 31). Additionally, in our study, drug adherence will be evaluated by the “pill count” method, in which the number of returned drugs will be counted. Drug adherence by pill count may be more accurate than the drug score (37, 38), and this is a strength of our study compared with previous studies. In addition, we will evaluate the concordance of drug adherence by pill count measurements or app-calculated indices.

## Ethics statement

The studies involving human participants were reviewed and approved by the Institutional Review Board of Seoul National University Bundang Hospital (B-2021-661-301). The patients/participants provided their written informed consent to participate in this study.
