# Supplementary material for: The ReInforcement of adherence via self-monitoring app orchestrating biosignals and medication of RivaroXaban in patients with atrial fibrillation and co-morbidities: a study protocol for a randomized controlled trial (RIVOX-AF)
Source: Front Cardiovasc Med. 2023 May 24;10:1130216. doi: 10.3389/fcvm.2023.1130216 (PMC10263056; doi:10.3389/fcvm.2023.1130216)

***Supplementary Material***

1. **Supplementary methods**

**Introduction**

Drug Adherence is a critical component of effective medical intervention, compliance to which ensures an optimal outcome, in line with the physician’s expectations can be achieved. Once a physician has determined the true adherence behavior of the patient, they can then utilize this information to apply effective treatment plans, especially in terms of continuing the present course of treatment or changing the recommendations, medicines, or the patient’s general wellness behaviors. However, acquiring this information in a trusted manner is a cause of concern. Generally, the patient’s adherence experience can be measured in two ways. Firstly, questionnaires and surveys form the first party source (i.e. patients) can provide a basic feedback, although the veracity of this information remains questionable. This mechanism is the most frequently applied one, however, due to the nature of many chronic diseases and general human behavior to comply with scenarios’ producing the best outcome, this sort of evaluation is subjective by nature. Secondly, application of technology assisted solutions, can be used to collect indirect information about the adherence behavior, which by themselves collect objective data. However, the ability of medical devices to collect the needed information for building a comprehensive picture of patient’s adherence behavior remains a major drawback. In the past, simple technological solutions, relying on the temporal information of patient’s acquiring drugs was used to determine the adherence behavior. However, these solutions are unable to determine the correct dosage units, counting inaccuracies, and the medication intake activity.

State-of-the-art tools and technologies are now aiding the design and development of holistic solutions. In particular, Artificial Intelligence assisted drug adherence monitoring solutions are now becoming a reality. To this end, we have developed a comprehensive, safe, and stable platform, which can manage the various intricacies of the drug adherence monitoring process, in a reliable manner. In support of this pilot study, this platform, with its modular architecture and multi-modal user interfaces, creates a communication channel between the healthcare providers and the patients.

Leveraging the computational and automation benefits of various tools and technologies, this platform provides a cohesive foundation for integration of AI based tools and information systems. Thus the physician, through a web interface, and the patient through a mobile interface can exchange appropriate subjective and objective information related to the drug adherence monitoring in real-time manner. Resultantly, both the physicians and the patients can be well-informed in terms of the applied treatment plan and necessary actions that can lead to good management of chronic and acute diseases.

**Motivation**

The motivation behind the development of this platform are three-fold.

1. Design and develop a platform to collect multi-modal data
2. Automate and simplify the process of monitoring drug consumption
3. Provide the physician with real-time, comprehensive data pertaining to the patient’s compliance and wellness markers.

Firstly, the platform has been designed to collect multi-modal data using various device integrations and AI assisted technologies. Resultantly, the patient data collected by this platform includes, biomarkers such as the pulse, blood pressure and weight of the patient, which are collected by third party devices integrated into the platform. The data is collected via Bluetooth interfaces, however, this data collection is not limited to only device based form. In fact, the patient can provide the necessary information by filling in the values, providing subjective information via surveys.

Secondly, since the users of this platform tend to be among the older populous, the mechanism to collect biomarkers and drug adherence data have been largely automated using AI assisted technologies. This alleviates unnecessary complications that arise from the use of latest technologies and provides the patients with a piece of mind. Processing the correct drug, as prescribed by the doctor, and identifying the drug intake is based on images taken in real-time by the mobile phone. The results are processed using small specific Machine Learning (ML) based models which can quickly determine if the patient is correctly complying with the physician’s directions or not.

This data collection is however, only part of the solution. This is where the third motivation comes into play, whereby the data is converted into information which provides the physician with the necessary tools to determine if the current treatment plan is working as anticipated or if a change is required. The information should include a quick overview of the patient’s compliance by determining a score for drug adherence and color coding the results and charts plotting the change in biomarkers over time. Additionally, once requested, detailed information on how and what data was collected and how it was converted into information should also be made available to the physician.

**System architecture and structural workflow**

The crux of adherence platform is performed on a dedicated server which provides access to the users in the form of a web application, to be used by the physicians, and a mobile application, which interfaces with the patient. The data collected from these User Interfaces(UI) is managed by the server application, which returns appropriate response messages and data, based on the customization and personalization features preset by the user. From a physician’s perspective, the drug adherence monitoring platform, provides a web based application to not only add new drugs and prescriptions for a patient but also follow, in real-time, the patient’s adherence behavior and to alter the treatment plan at any point. On the other hand, the patient operates a mobile application, which provides timely notifications for the patient to consume a drug and also helps to identify the correct drug and its dosage using AI based medication recognition, and determines if the patient is actually taking the drug, again using AI based facial action recognition. Supplementary services, on the server side, reduce the computing and storage cost for the end user and provide platform specific services, such as authentication and authorization, patient management, drug adherence time and intake manager, and many others. The main components of the architecture are the Manager which interfaces with the Drug Adherence Web application and the Dug Adherence Mobile application to collect prescription and intake data respectively. Correspondingly, the information shared with the mobile application is in the form of intake notifications and compliance messages, while for the web application it includes patient adherence information for drug intake and biomarkers. The reasoners, process the input data to identify the appropriate rules, which should be fired and the conclusions which should be utilized for information processing. The service component contains the business logic, which applies internal processing on the data and prepares it for storage or as a response to the user query. The storage handler, controls the conversion of data into appropriate form for the data store. These services encapsulate a MySQL datastore which is used to persist the various features and data necessary to enable the drug adherence process.

Together, these modules work in cohesion to collect input from the physician and the patient and to create information which is used by the opposing stakeholder for compliance and analysis (Figure 1). Further details of these modules, follows as under.

1. **Manager**

The Manager module is the primary interface between the server application and the user interfaces. This module, collects the requests coming from external devices and process the response from internal modules, to convert them into a form consumable by the target service and device, respectively.

Once the physician observes a patient in the real world, they can provide details of the drugs and their prescription for a patient, within a period, which is used to create a prescription for the patient. This prescription information is sent to the Drug Adherence Time Manager, which manages the state of prescription for each patient. In addition to the information on the patient and the drug, a prescription also holds necessary artifacts to identify the period over which it is applied, drug repetition per day and per week, and the time when the patient should be notified to take the drug. This information is used by the server application to prepare alarms for the patient, to take the drug at the pre-set time.

Once a patient, receives an alarm on the mobile application, they can start the intake process for Drug Adherence. Here, two key services are executed to automate the process of identifying the correct drug and to ensure that the patient takes the drug. On the mobile application, these services utilize the built-in camera, to take pictures of the drug and the intake process. These pictures are sent to the server to process and determine if the patient is taking the correct drug and is placing the drug in their mouth or not.

1. **Reasoner**

The reasoner is the core component of this platform which comprises Face Recognition Reasoner and Intake Reasoner, where the latter one comprises Medication Recognition Reasoner and Action Recognition Reasoner (Supplementary Figure 1 and 2).

**Face Recognition Reasoner**

Two different processes are performed, sign up and sign in.

- **Sign up process**: At first, the app activates webcam to capture a picture within the camera’s field of view, wherein user’s face and/or upper body with surrounding background may be available. Subsequently, the captured image is sent from the smartphone to the dedicated server through the internet. It is worth noting that otherwise specified, all the operations of AI models in later processes take place in server side. Accordingly, in the server, the RetinaFace model (1) using deep learning technique is adopted to execute the face detection operation. If there is no face detected, the app shall perform the screen of automatic picture capture again; otherwise, the target region of user’s face, which corresponds to the existence of strong predefined facial features inside the image, can be localized and then cropped. Afterwards, the step of facial feature extraction using another deep learning architecture, namely FaceNet (2) is activated to extract features from the detected face. The acquired feature vector is finally stored to a predefine user authentication database, wherein a key-value pair representing the encoded user ID and the facial features (in form of high-dimensional feature vector) is generated.
- **Sign in process**: The same sequential process of smartphone’s front camera activation, automatic picture capture, face detection, and facial feature extraction as mentioned in the sign up process is performed. Once the considered user’s facial feature is extracted by the FaceNet, cosine similarity measure is adopted to pairwise compare the considered facial features with all of the counterparts stored in the user authentication database, from which the user ID corresponding to the highest similarity score is returned. Then, a verification pop-up is displayed to double check whether the returned ID belongs to the current end-user or not. If he/she confirms, user authentication is successful; otherwise, a pop-up notification of facial login failed appears and then the app returns to the screen of automatic picture capture.

**Medication Recognition Reasoner**

At first, the app activates the smartphone’s back camera to automatically capture a picture within the camera’s field of view, wherein certain objects with surrounding background (e.g., opening hand’s palm, surface, desk/table, etc.) may be available. Next, the MobileNetV2 backbone with SSDLite detection head, a type of deep learning architecture, is adopted to produce bounding boxes surrounding all of the salient objects inside the captured image. To this end, if there is no object detected, the app shall perform the screen of automatic picture capture again; otherwise, the detected object, which corresponds to the existence of strong saliency inside the image, can be localized and then cropped by the inferred coordinates the above-mentioned bounding boxes. Then, all of the images of detected objects are respectively fed into another deep learning model called EfficientNetB2 (3) pretrained for medication classification due to its powerful representations of pill image features (e.g., shape, color, etc.). Remarkably, to train this model, we generate a specialized dataset consisting of five categories, 3,600 non-medication images randomly selected from ImageNet dataset (4), 3,600 for each type of rivaroxaban pills among totally four different types (note that the amount of front-sided and back-sided samples are equally created). We have used Samsung Galaxy S7’s back camera to collect RGB images of these pills with varied resolution (given maximum height of 500 and maximum width of 500) and under diverse background common in practice such as various desk’s texture, colorful matt, hand’s palms, etc. Then, the original images are augmented by different levels of brightness, contrast, and sharpness. In addition, softmax cross-entropy objective function and gradient descent optimizer is applied to train the medication recognition model for this application. In the next step, if the user takes the correct pill, smartphone’s back camera is terminated and the medication intake detection stage is subsequently proceeded; otherwise, which means the user takes picture of wrong pills, the process returns to the step of automatic picture capture.

**Action Recognition Reasoner**

To this end, smartphone’s front camera is activated to automatically capture the user’s face inside the surrounding environment. Next, the RetinaFace mode is executed again to detect user’s face. If there is no face detected, the app shall perform the screen of automatic picture capture again; otherwise, the target region of user’s mouth-related landmarks, which corresponds to the existence of strong predefined mouth-based features inside the face image, can be localized. Particularly, landmarks of the bottom of upper lip and the top of the lower lip are retrieved from the image represented in the detected face. Afterwards, the vertically corresponding points of these two parts are used to compute the average distance between upper and lower parts of the considered lip for the consideration of mouth-opened action. Consequently, if the result distance value is smaller than a pre-defined threshold value, the patient is considered as opening the mouth. Otherwise, a pop-up notification of guiding the patient to open the mouth appears to meet our requirement of medication intake. Furthermore, in the region covered by considered lips-related landmarks, the aforementioned procedure of object detection (using MobileNetV2 backbone with SSDLite detection head) followed by medication classification (using EfficientNetB2) is re-utilized to examine whether any medication exists within that region of interest and then again verify if that to-be-taken pill is the right one. If so, the smartphone’s front camera is terminated and the screen of medication intake successful appears, which marks the completion the medication recognition and intake detection stages.

1. **Services**

Business logic, related to processing the external request and preparing appropriate responses is handled by the services module. Here, these services are of particular interest. Firstly, the Patient Service, executes various operations to manage the activities related to the patient. These include operations to create a new patient, update the record of an existing patient, and fetch the partial and complete record of the patient. The results of these operations are customized based on the user making the original request. Thus if a physician, belong to a particular hospital requests patient data, only the records for patients, belonging to the particular hospital are returned. Similarly, the operations to enable or disable a patient, by an administrator is also provided by this service.

Secondly, the Drug Service, manages the drugs present in the server application. The operations present in this service can be used to not only create a new drug instance but also to collect record for all patients or prescriptions, associated with a particular drug. This service is also able to dynamic alter the priority and label of a drug which is used in the intake identification process to automatically customize the output of the machine learning model in terms of the drug label and priority.

Thirdly, the Prescription Service is used to create the generic prescription, which is then converted into alarm instances, each of which represents a particular time in which the prescription should be taken. While the generic prescription only holds information on which drug is to be taken, the day of week, and time of the day when it should be taken, the alarm instance is an atomic unit of when and what related to the prescription. As an example, if the prescription is set to 01/12/2022, repeat twice every Friday, at 06:00am and 04:00pm, then two alarms will be generated for 02/12/2012 (Friday) at 06:00am and 02/12/2012 (Friday) at 04:00pm. At these particular times, an alarm notification is sent to the mobile app, which informs the user to take a particular drug.

1. **Storage Handler**

The Storage handler, provides an abstraction on the datastore, to resolve object-relational impedance mismatch and enable the services to run on the object models, while the data is stored in relational model. The object models are described in the Object-Relational Mapping module, which also controls the creation and management of the database. The models contain variable and programming specific datatypes which are managed and translated by the Hibernate ORM into database specific format. All models also hold a primary key, which uses Universally Unique Identifier (UUID) for the users and Long type identifiers for the other entities. Additionally, the relationships between the objects are translated into associated tables and foreign key based relationships in the databases. This is particularly important for simplifying one-to-many and many-to-many relationships which are translated into simple object maps which are easy to use for abstract level programming. Additionally, each model also contains setter and getter methods to provide appropriate encapsulation of the data elements and to provide a consistent interface for manipulating them.

The Data Access Object (DAO) Manager, provides the operations to apply Create, Retrieve, Update, and Delete (CRUD) operations on the data models to manage their state in the data store. Since the DAOs implement the same abstract interface, they contain two methods for object retrieval, including a single instance retrieval using the id and multiple object retrieval for the model. DAOs are accessible only by the services and can be used to finally commit the changes into the data store.

1. **Datastore**

The datastore used for this application is the MySQL Relational database. In this type of database, relations (tables) represent the entities which contain the data pertaining to instances, as tuples. Each tuple is identified by a unique identifier, also known as the primary key. A database entity can be linked to another entity using the foreign key relationship. The foreign key relationships are of two kinds, with explicit foreign keys, being registered into the database management system and used for enforcing rules at the database level, while implicit foreign keys are managed by the abstract layer on top of the database and is responsibility of the business logic in the service layer. Due to the use of ORM, the database remains safe from inconsistencies and incorrect operations, which may render the data corrupt, otherwise. In this way, we ensure that the data is kept safe in the database. Additionally, since access to this database can only be achieved by the abstract server application above it, it also keeps the user secure from malicious attacks.

***System Implementation Outcome: Process Flow***

The system comprises two apps: one is a mobile app (Drug Adherence mobile app) which is a patient-oriented app while the other one is a web app (Drug Adherence web app) which is a physician-oriented app (Figure 2 and Supplementary Figure 3). Each app has their own process where web app is used for new patient registration and prescription while mobile app is used for drug adherence by the patient.

**Patient Registration and Prescription Process**

- **Step 1**: The physician logs in to the web app with their account. They can only check the patients belonging to their affiliation. New patients will be automatically affiliated to the according hospital.
- **Step 2**: Patients can take picture via webcam if they want to enable facial login. Otherwise, this process can be skipped as it is not mandatory. Profile information is added which are name, gender, date of birth, email address, patient’s account ID and password.
- **Step 3**: Entering to view visit screen on individual patient, the visit slot is appeared on top showing start date and end date for each visit. In the bottom side, drug adherence statistics are shown in overall, daily and weekly adherence graph.
- **Step 4**: Entering to drug adherence calendar screen, calendar is shown with drug adherence status. Adherence time appears with colored circle on each day. Blue colored circle is showing the upcoming schedule, green colored circle means the medication is taken correctly while the red colored circle means the patient has not taken the medication.
- **Step 5**: Entering to administer rivaroxaban screen, physician inputs the necessary information such as the type of rivaroxaban, time to intake, the period, and the end date. Pressing the complete button will return the screen back to drug adherence calendar, showing the newly added adhere schedule.

**Medication Recognition Process**

- **Step 1**: The patient logs in the app with either manual input or facial recognition. Account information can be given by the physician during the new patient registration stage in the web app. Facial recognition will only work for those who have registered their face. To prevent the mistake of recognizing similar looking patient, a prompt with the patient name appears to check if it’s the right patient.
- **Step 2**: The main screen of the app shows the drug to take and times. In here, the patient can change the alarm type to either sound or vibration. Patient can also change the alarm time with their own preference. There are two additional buttons, complete medication intake and start recognition process. Complete medication intake button is used when the user has forgotten to perform the recognition process or two hours has passed from the preset time. Start recognition button can be used when the user is unable to proceed the recognition process on time, so to proceed recognition in advanced.
- **Step 3**: When the preset time comes, alert screen is shown. There are two different situations, when the screen of the smartphone is off or on. If the screen is off, the sound will ring or vibrate. If the patient turns on the screen, he/she will enter to the alert screen. When the screen is on, typical android notification will appear on the screen and will enter to alert screen when pressing it. There are two buttons in the alert screen, cancel or proceed. Proceed will enter to medication recognition screen while cancel button will cancel the alarm. Then the alarm will ring again on the time of next cycle.
- **Step 4**: In medication recognition screen, smartphone’s back camera is activated to take the picture of the pill. Other screens are masked except the middle square where the pill should be located inside that boundary. If an object is not detected or the pill is not the right one, it will show a toast message and will repeat whole cycle again.
- **Step 5**: Once medication recognition is successfully done, smartphone’s front camera is activated to proceed action recognition. This will first detect the face, and then detect the mouth whether it is opened or not. If the mouth is not opened over the specific threshold, a toast message is shown to open the mouth. Once the mouth open is detected, same procedure is proceeded as in medication recognition stage. If this is failed, the whole cycle will repeat again.
- **Step 6**: In Drug adherence screen, adherence calendar is shown, similar in the web app. Due to the narrowness of the nature of the smartphone screen, only colored circle is shown on each day. By clicking each day, adherence time appears on the bottom of the calendar. Three additional buttons are in the bottom side, view overall adherence, view daily adherence, view weekly adherence, respectively.
- **Step 7**: In overall adherence screen, overall adherence status is shown in pie chart. Daily adherence screen shows the daily intake status in scrollable bar chart. Similarly, Weekly adherence screen shows the weekly intake status in scrollable bar chart where patients can check their dug adherence status by themselves.

**References**

1. Deng J, Guo J, Ververas E, Kotsia I, Zafeiriou S, editors. Retinaface: Single-shot multi-level face localisation in the wild. *Proceedings of the IEEE/CVF conference on computer vision and pattern recognition*; 2020.

2. Schroff F, Kalenichenko D, Philbin J, editors. Facenet: A unified embedding for face recognition and clustering. *Proceedings of the IEEE conference on computer vision and pattern recognition*; 2015.

3. Tan M, Le Q, editors. Efficientnet: Rethinking model scaling for convolutional neural networks. *International conference on machine learning*; 2019: PMLR.

4. Deng J, Dong W, Socher R, Li L-J, Li K, Fei-Fei L, editors. Imagenet: A large-scale hierarchical image database. *2009 IEEE conference on computer vision and pattern recognition*; 2009: Ieee.

**Supplemental Figure 1. Workflow of face recognition sign up and sign in process**


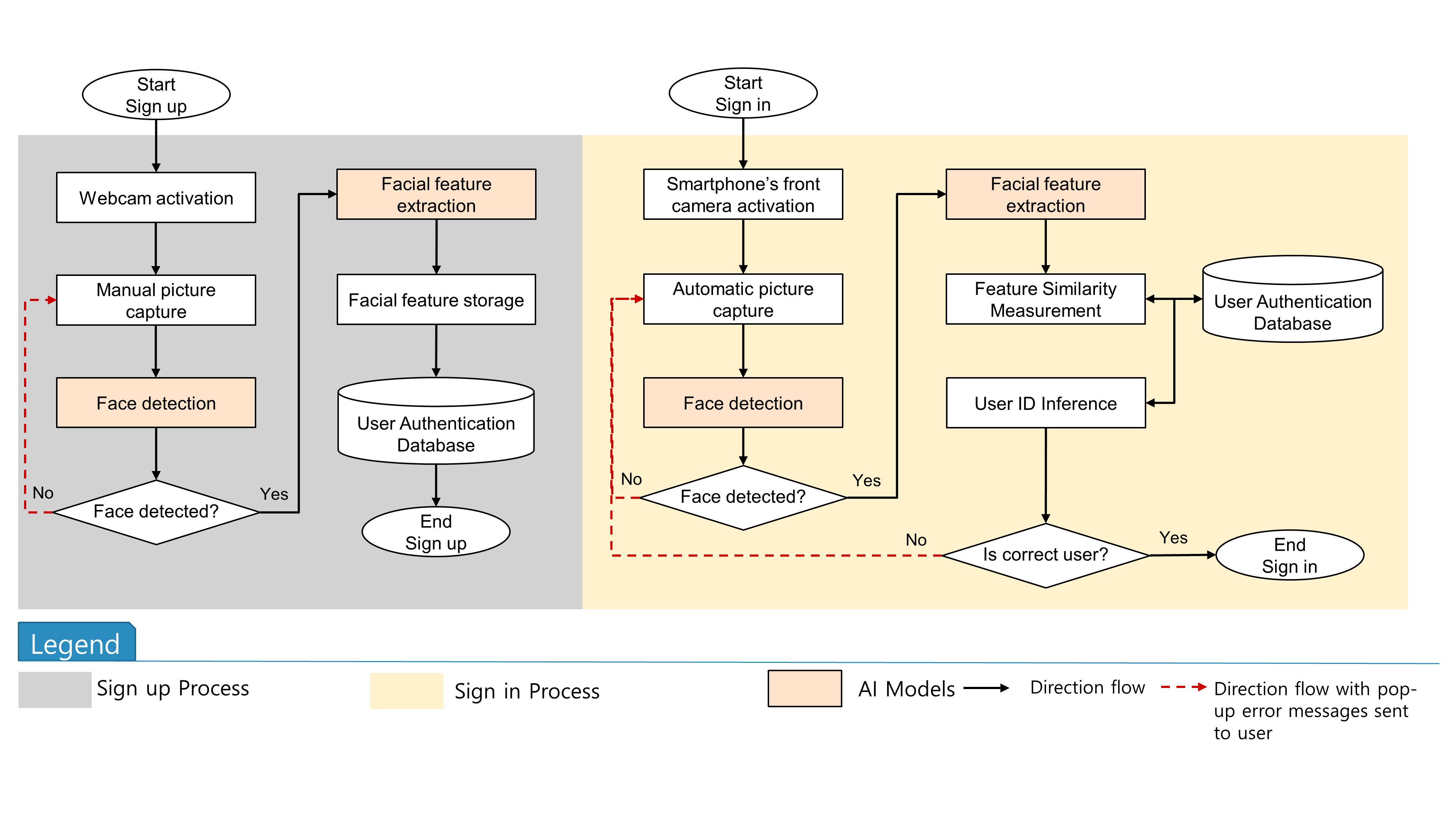


**Supplemental Figure 2. Workflow of medication recognition and action recognition process**


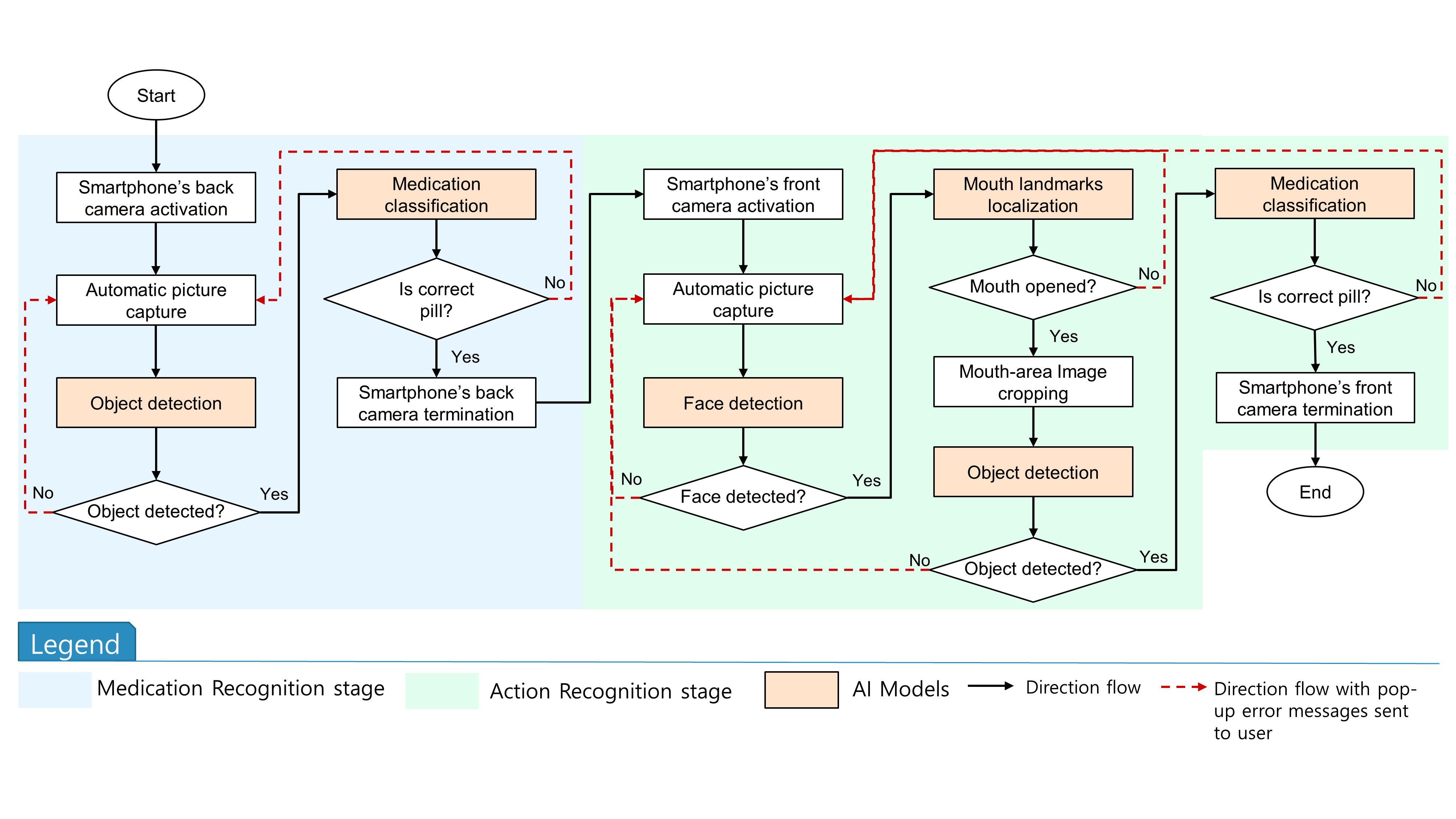


**Supplemental Figure 3. Patient registration and dashboard system**


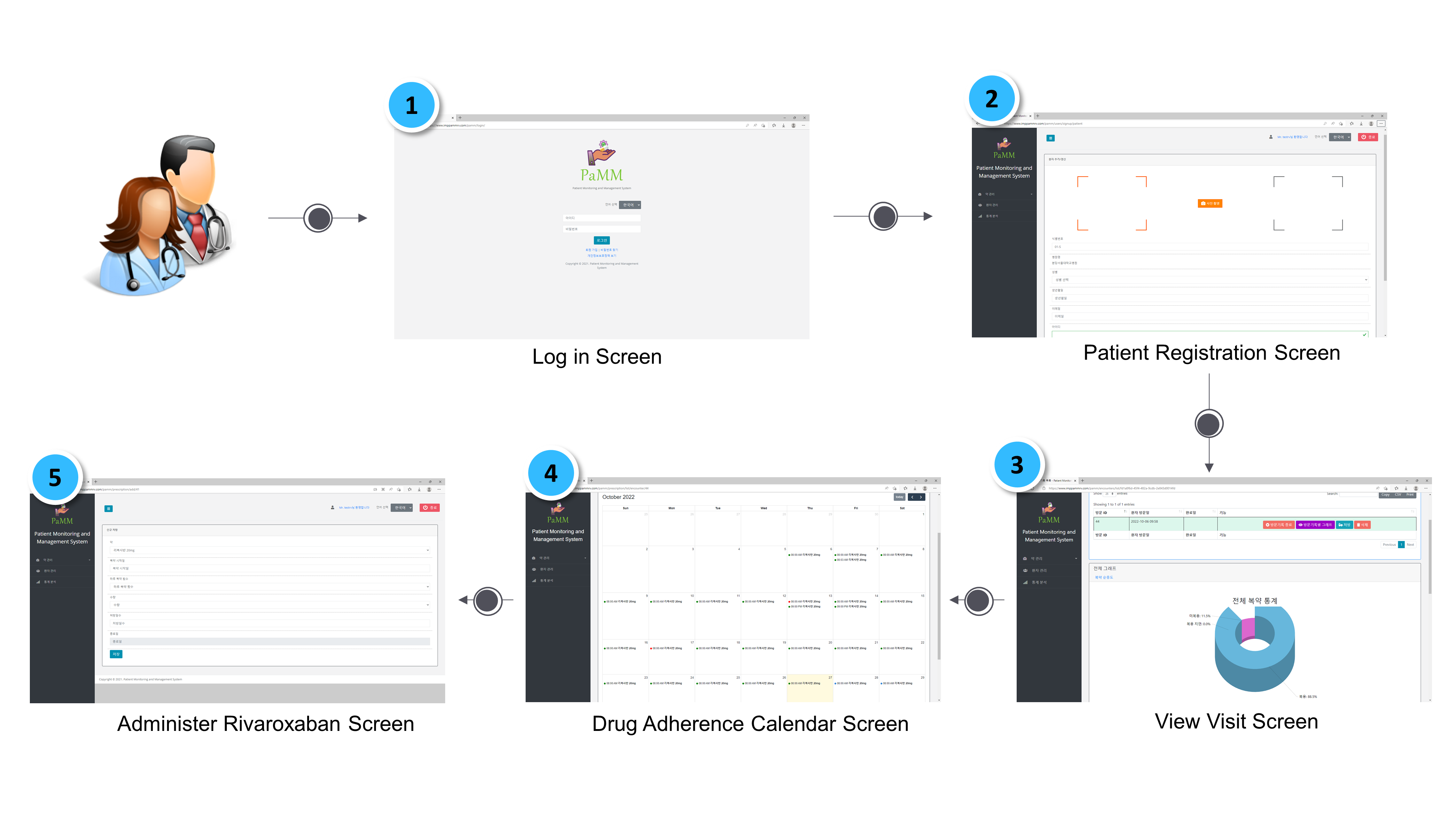

Supplement: Supplementary file 1 [file Table2.docx]
